# Influence of muscle fitness test performance on metabolic risk factors among adolescent girls

**DOI:** 10.1186/1758-5996-2-42

**Published:** 2010-06-23

**Authors:** Jorge Mota, Susana Vale, Clarice Martins, Anelise Gaya, Carla Moreira, Rute Santos, José C Ribeiro

**Affiliations:** 1 Research Centre in Physical Activity, Health and Leisure, Faculty of Sports - University of Porto - Portugal

## Abstract

**Background:**

The purpose of this study was to examine the association between muscular fitness (MF), assessed by 2 components of Fitnessgram test battery, the Curl-Up and Push-Ups tests and the metabolic risk score among adolescent girls.

**Methods:**

A total of 229 girls (aged 12-15 years old) comprised the sample of this study. Anthropometric data (height, body mass, waist circumference) were collected. Body mass index (BMI) was also calculated. Muscular strength was assessed taking into account the tests that comprised the FITNESSGRAM test battery, i.e. the curl-up and the push-up. Participants were then categorized in one of 3 categories according the number of tests in which they accomplished the scores that allow them to be classified in health or above health zone. The blood pressure [BP], fasting total cholesterol [TC], low density lipoprotein-cholesterol [LDL-C], high density lipoprotein-cholesterol [HDL-C], triglycerides [TG], glucose, and a metabolic risk score (MRS) were also examined. Physical Activity Index (PAI) was obtained by questionnaire.

**Results:**

Higher compliance with health-zone criteria (good in the 2 tests), adjusted for age and maturation, were positive and significantly (p ≤ 0.05) associated with height (r = 0.19) and PAI (r = 0.21), while a significant but negative association was found for BMI (r = -0.12); WC (r = -0.19); TC (r = -0.16); TG (r = -0.16); LDL (r = -0.16) and MRS (r = -0.16). Logistic regression showed that who were assigned to MF fittest group were less likely (OR = 0.27; p = 0.003) to be classified overweight/obese and less likely (OR = 0.26; p = 0.03) to be classified as having MRS. This last association was also found for those whom only performed 1 test under the health zone (OR = 0.23; p = 0.02).

**Conclusions:**

Our data showed that low strength test performance was associated with increased risk for obesity and metabolic risk in adolescent girls even after adjustment for age and maturation.

## Introduction

A lack of physical fitness (PF) has been associated with the development of cardiovascular disease risk factors (CVD) in youth, such as lipid disorders, high blood pressure, insulin resistance among others [1]. This is a cause of concern because CVD risk factors tend to track to adulthood [2] and the process of atherosclerosis starts early in childhood [3]. Conversely, some studies have shown that PF levels track from childhood to adolescence [4] and from adolescence to adulthood, [5,6] with moderate to strong coefficients for cardio-respiratory fitness (CRF) and strength, respectively. [7,8] Additionally, some data pointed-out that in adolescents PF and especially CRF has been decreasing over time [9,10] Thus, PF was proposed as a major marker of health status at any age [1]. Given this figure, which affects an increased number of children, [11,12] poor PF represents a health problem ought to be screened [13].

In spite health-related PF variables included CRF, muscular fitness (MF), namely abdominal muscular strength and endurance, lower back flexibility and fatness, [14] studies in youth have mostly looked at the associations between CRF and CVD [15,16]. Further, the majority of the studies addressing MF linked it with fatness. For instance, it was shown an association between MF and central and whole body composition in normal -weight [17,18] as well as overweight children [17]. Other studies showed that muscular strength was identified as an independent and powerful predictor of better insulin sensitivity in children [19]. However, a recent review showed that due to a limited number of studies, inconclusive evidence emerged for a relationship between muscular strength or motor fitness and CVD risk factors [20]. Therefore, there is scarce information about the association of muscular fitness with, either, individual or clustered CVD risk factors. However, strength and muscular fitness have been recognized in the prevention of both adults [21] and elderly [22] diseases and functionality. Furthermore, strength has gained importance regarding health-related recommendations through exercise that increase bone health [23].

Additionally, gender differences have been stressed. Indeed, girls seem to be a key targeting group in health-related PA promotion. It is known that PF has a strong association with physical activity (PA) and reviews of correlates of PA in youth showed that one of most important variables consistently associated with PA was gender, with studies suggesting a consistent physical activity level decline with increasing age and tend to be lower in girls than boys [24].

In this context, the purpose of this study was to examine the association between muscular fitness, assessed by two components of Fitnessgram test battery, the Curl-Up and Push-Ups tests with clustered metabolic risk among adolescent girls.

## Methods

### Participants

A total of 229 girls (aged 12-15 years old) comprised the sample of this study. **As exclusion criteria girls were asked if they have some physical constraint or medical conditions that do not allow them to participate on this study. Thus**, girls that participated in this study were apparently healthy and free of medical treatment and they were living in Porto District. A letter informing families that students would be measured was sent home two weeks before measurements took place each year. Schools approved the study protocol and all parents signed an informed consent form. All measures were carried out by the same group (Physical Education teachers, medical doctor and nurse). This study was approved by the Foundation for the Science and Technology of the Portuguese Ministry of Education. All students were invited to perform a fitness battery tests and to answer a questionnaire. Fitnessgram test battery is included in the national curriculum; however participation was voluntary for all evaluations.

### Daily Evaluation protocol

In relation to the daily measurement protocol, girls were firstly identified through her code number. Secondly, blood samples were taken followed by blood pressure measurements. Girls were then given breakfast followed by the determination of their maturational stage. Finally the Fitnessgram tests were performed. The variables were measured between 8:00 and 11:00 am.

### Anthropometric Measures

Anthropometric methods were used to measure body mass and body height. Body height was measured to the nearest mm in bare or stocking feet with girls standing upright against a Holtain Stadiometer. Body mass was measured to the nearest 0.10 kg, lightly dressed and after having breakfast, using an electronic weight scale (Seca 708 portable digital beam scale). Body mass index (BMI) was calculated from the ratio of body weight (kg)/body height (m^2^) and organized using age and sex adjusted cut-off points described by [25]. Then girls were categorized as normal weight, overweight or obese group. However, for analytical purposes the overweight and obese girls were collapsed in one group, the overweight/obese one.

Waist circumference (WC) was measured at the superior border of the iliac crest and was further used in the derivation of the metabolic syndrome risk score.

### Blood sampling

Capillary blood samples of participants were taken from the earlobe after at least 12 hours fasting in order to obtain values of plasmatic total cholesterol (TC), high density lipoprotein cholesterol (HDL), triglycerides (TG), and fasting glucose (GLUC). The lipid profile does not measure LDL level directly but instead estimates it using the Friedewald equation: LDL cholesterol = total cholesterol - HDL cholesterol - (0.20 × triglycerides) [26]. The blood samples were drawn in capillary tubes (33 μl, Selzer) coated with lithium heparin and immediately assayed using Colestech LDX Analyser where a unique system on the cassette separates the plasma from the total blood from the blood cells. The Cholestech LDX^® ^cassettes were stored in the refrigerator after reception. They were put at room temperature 10 minutes before opening and then were immediately used. Expiration day was checked before use. A daily optic check was performed on the analyser used for the study. The Cholestech LDX^® ^analyser provide good agreement with laboratory measures for population-based screaming for cardiovascular risks factors. [27] Additionally, it meets the criteria set by lipid standardization panel for accuracy and precision of cholesterol measurements [28].

### Blood pressure

Blood pressure (BP) was measured using the Dinamap adult/pediatric and neonatal vital signs monitors, model BP8800. Measurements were taken by a trained technician and with all children sitting after at least 5 min rest. Two measurements were taken after five and ten minutes rest. The mean of these two measurements was used for statistical analysis. If the two measurements differed by 2 mmHg or more the protocol was repeated (two new measurements until the difference did not exceed 2 mmHg), which was already previously used in a similar population [29].

### Metabolic Risk Score (MRS)

Given the lack of a universal definition for metabolic syndrome in children and adolescents [30] we decided to compute a continuous risk score from the following measurements: TC; HDL-C; LDL-C; TG; glucose; systolic blood pressure (SBP) and the WC, similarly to what has been proposed by Andersen et al [31]. For each of these variables, a Z-score was computed as the number of SD units from the sample mean after normalization of the variables, i.e., Z = ([value - mean]/SD). The HDL-C Z-score was multiplied by -1 to indicate higher CVD risk with increasing value. Z scores by age were computed for all risk factors. Then, MRS was constructed by summing the z scores of all individual risk factors. A lower metabolic risk score is indicative of a better overall CVD risk factor profile. To calculate the odds ratio, metabolic risk was dichotomized at the cut-off value plus 1 SD, identifying children with cluster risk. High metabolic risk was considered when the individual had 1 or more than 1 SD of this score.

### Biological maturity

Regarding the maturational stage, girls were inquired separately during physical examination. Each girl self-assessed her stages of secondary sex characteristics. Stage of breast was evaluated according to the Tanner's criteria. Previous study showed a correlation of 0.73 between ratings on two occasions (three day interval) in a sub-sample of 50 selected subjects [32]. In that study, concordance between self-assessments of sexual maturity status and physician assessment was 63% for girls.

### Muscle Fitness Assessment (MF)

MF was assessed taking into account the strength tests that comprised the FITNESSGRAM test battery, i.e. the curl-up and the push-up [33]. The Curl-Up test was used to evaluate abdominal strength. Girls lie down with knees bent and feet unanchored. Set to a specified pace, students complete as many repetitions as possible to a maximum of 75. The Push-Up test was used to evaluate upper body strength. Girls lower body to a 90-degree elbow angle and push up. Set to a specified pace, students complete as many repetitions as possible. In the present study, test-retest reliability over one week (n = 50) ranged between r = 0.91 for curl-up and r = 0.87 for push-up. The *Fitnessgram *is included on Physical Education curriculum of Portuguese National Program. All the physical education teachers involved in this project undertook training sessions, worked together each year, with qualified staff in order to assure the standardization, and reliability of the measurements. Students were familiarized with the procedure for each test before data collection. Further, the participants received verbal encouragements from the investigators in order to achieve maximum performance. The scientific rationale to select these tests, as well as their reliability in young people, has been previously published [33].

Analysis was conducted including the percentage of students meeting the adopted age-adjusted criterion referenced health standards (Health Fitness Zone) for each MF test item in the Fitnessgram test battery, which allow us to assigned, for each test, girls as belonging to a healthy zone (fit) or under a healthy zone (unfit). Thus, based upon their individual performance in each MF test, girls were assigned to 3 groups. The MF0 (n = 37; 16.2%), which comprise those who did not reach the healthy-zone in both MF tests; the MF1 (n = 90; 39.3%), which comprised those girls who reached at least one score within the health-zone in both tests and, the MF3 (n = 102; 44.5%), which comprised those girls whom reached the health-zone standard in both MF tests.

### Assessment of Physical Activity

Physical Activity was assessed by a questionnaire previously used, which showed good reliability (ICC: 0.92 to 0.96) [34]. The questionnaire has five questions with four choices: **(a) **- Outside school do you take part in organized sport?; **(b) **- Outside school do you take part in non-organized sport?; **(c) **- Outside school, how many times a week do you take part in sport or physical activity for at least 20 minutes?; **(d) **- Outside school hours, how many hours a week do you usually take part in physical activity so much that you get out of breath or sweat?; **(e) **- Do you take part in competitive sport?

Overall a maximum of 20 points can be reached. A PA index was obtained, which divided the sample into four different activity categories, according to the total sum of the points: the sedentary group (0-5); low active group (6-10); moderately active group (11-15) and vigorously active group (16-20). However, for statistical analysis girls were grouped into two categories: the less active group (LAG), which included both sedentary and low active youth; and the active group (AG), which included the moderately active and the vigorously active group.

### Statistical Analysis

Descriptive data are presented as means and standard deviation. All variables were checked for normality and appropriately transformed if necessary. Differences across MF (MF0, MF1 and MF2) groups for individual CVD risk factor variables were assessed by analysis of covariance (ANCOVA), controlling for maturation and age. Posthoc analyses were analyzed by the Bonferonni multiple comparison tests. Logistic regression analysis was used to analyse the influence of MF on BMI (normal vs. Overweight/obese) - model 1, physical activity (active vs. Low active) - model 2 and MRS (yes or no) - model 3.

The level of significance was set at p ≤ 0.05. Data were analyzed using SPSS (Windows version 15.0).

## Results

Descriptive statistics for the CVD risk factors across the MF groups are presented in table 1. Girls with better MF profile (those assigned to MF2 group), where significantly taller, had low BMI, had better lipid profile (TC, TG), were more active and had a better MRS than their less fit (MF0) counterparts, after adjustments for age and maturation.

**Table 1:** Descriptive characteristics of the participants

	MF0	MF1	MF2
	(n = 37;16.2%)	(n = 90;39.3%)	(n = 102; 44.5%)
Age, years	13.9 ± 1.9	14.7 ± 1.9	13.8 ± 2.0
Weight (kg)	51.6 ± 9.8	55.0 ± 11.1	54.1 ± 9.6
Height (cm)	154. 3 ± 8.9	158.4 ± 6.5	160.1 ± 7.2*
BMI (kg/m²)	21.7 ± 3.7	21.8 ± 3.6	20.9 ± 2,7*
WC (cm)	76.6 ± 10.0	76.2 ± 8.6	73. ± 7.4*
SBP (mm Hg)	119.1 ± 12.0	121.3 ± 13.2	124.5 ± 13.2
DBP (mmHg)	63.2 ± 7.5	64.9 ± 8.8	66.1 ± 9.2
TC (mg/dl)	157.5 ± 23.2	152.3 ± 26.7	151.1 ± 26.4*
HDL (mg/dl)	43.8 ± 14.2	46.9 ± 10.1	46.7 ± 11.1
LDL (mg/dl)	100.3 ± 20.8	93.8 ± 24.5	89.7 ± 26.3
TG (mg/dl)	66.8 ± 26.2	59.9 ± 18.4	57.4 ± 17.8*
Glucose (mg/dl)	83.1 ± 8.7	83.2 ± 6.5	84.6 ± 6.8
MRS	1.57 ± 4.2	0.13 ± 2.6	-0.39 ± 3.2*
PAI	9.8. ± 3.4	10.1 ± 3.7	11.6 ± 3.9*

Data are means ± SD. ANCOVA's Test adjusted for age and maturation, with Bonferroni corrections: * p < 0.05 - significantly different from MF0. MF - muscular fitness; PAI, physical activity index; TC, total cholesterol; TG, Triglycerides,

Partial correlations for MF with individual and clustered CVD risk factors are shown in table 2. Higher compliance with health-zone criteria (good in the 2 tests), adjusted for age and maturation, were positive and significantly (p ≤ 0.05) associated with height (r = 0.19) and PAI (r = 0.21), while a significant but negative association was found for BMI (r = -0.12); WC (r = -0.19); TC (r = -0.16); TG (r = -0.16); LDL (r = -0.16) and MRS (r = -0.16). No other statistical significant differences were found.

**Table 2:** Associations between MF and metabolic, anthropometric and physical activity, adjusted for age and maturation

Variables*	Height	BMI	WC	TC	TG	LDL	PAI	MRS
**MF**	**r = **0.19	**r **= -0.12	**r **= -0.19	**r **= -0.16	**r **= -0.16	**r **= -0.16	**r **= 0.21	**r **= -0.20
	**p = **0.01	**p **= 0.05	**p **= 0.008	**p **= 0.02	**p **= 0.03	**p **= 0.02	**p **= 0.004	**p **= 0.006

MF - Muscular Fitness; BMI - Body Mass Index; WC - Waist Circumference; TC - Total Cholesterol; TG, Triglycerides; PAI - Physical Activity Index; MRS - Metabolic Risk Score

* Only shown those that p ≤ 0.05

Logistic regression showed that those who were assigned to fittest group (MF2) were less likely (OR = 0.27; p ≤ 0.05) to be classified as overweight/obese and less likely (OR = 0.26; p ≤ 0.05) to be assigned to MRS group compared MF0 group. This last association was also found for those whom only performed 1 test (MF1) under the healthy zone (OR = 0.23; p = 0.02). No statistical significant associations were found with regard PA.

## Discussion

Since the onset of chronic disease risk factors lies in early childhood, [31,35] it is of great importance to examine the risk predictors in order that effective preventive strategies targeting those at risk start as early as possible.

The main finding of this study is that it shows a tendency for girls in the MF2 group to possess a better CVD risk factor profile compared with those with low MF (MF0). Further, our data showed that higher MF is associated to better MRS profile and low BMI after adjustments for age and maturation, which highlighted the potential importance of a good MF with regard metabolic risk factors.

These findings are worthy to comment in two ways. First, the majority of the studies used CRF to characterize associations between PF and individual or clustered CVD risk factors [36,37]. Those who used MF showed that low levels of abdominal strength and upper body strength were associated with higher BMI, [38-40] which agrees with our data. However, at best of our knowledge few studies have addressed the relationship between MF and other metabolic risk factors besides fatness. Our data clearly showed that those girls who were assigned to low fit group (MF0) were more likely to be classified at risk of having a MRS, which is in line with other study suggesting an inverse association between muscular strength and lipid metabolism profile [41]. Further, it should also be noted that in our study even girls that failed one health-zone-based test (MF1) had a protective association with regard MRS (OR = 0.33; p = 0.02). Although this study is based on a cross-sectional design our data emphasised the importance of MF as a potential factor in the prevention of further metabolic risk development. This issue was recently addressed in Norwegian students and data suggested that MF may have an independent protective factor in the development of CVD risk factors [42].

The second important comment lies on the idea that decreased muscular strength and power may affect the youth's ability to become skilled and successfully perform daily activities particularly during their development years [43]. Given the fact that girls are more likely to gain weight [44] and tend to be less active, [45] as they get older, a special attention in their development years is recommended. Indeed, our data also showed an inverse association between MF and BMI. Given the fact that usually obese are more prone to cluster the CVD risk factors [46,47] our data raises some further concerns. In fact, although puberty is the greatest confounding factor with regard the application of the metabolic syndrome (or clustered factors) in pediatric population our data showed significant associations even after adjustments for maturation. On the other hand, the association between MF and MRS seems to occur even in prepubertal stages [42] and, therefore, the role of MF needs to be further investigated. It was also suggested that MF may be a function of participation in PA [42]. Thus, reducing sedentary activities, increasing PA and PF levels may be required to protect youth from excessive weight gain as well as other metabolic diseases.

The main strength of this study was that the use of field tests for fitness assessment, showed an association between MF and MRS, which, in turn, stresses the importance of PF test application in the school system. Therefore, the schools, which commonly administer physical fitness tests, are prime sites for identifying high-risk children, in addition to sources for instituting specialized programs for these children. Further, adjustments for age and maturation gave additional strength to the data found. Limitations should be recognized. First this study is a cross-sectional design **with a relative small sample and only focus on girls**. The intent was to explore associations between MF and metabolic risk factors, **but it is not possible to inferred causal relationships with such a design and, therefore results should be looked with caution**. As a result, additional data is needed to replicate these findings using **both **longitudinal designs and **representative samples**. **Further this study would benefit from additional collected data such as combined socioeconomic position, which can enhance the outcomes**. Secondly either obesity or MF was assessed indirectly. In this study obesity was not considered on the basis of an objective measure of body composition. Although BMI has become a very common way of assessing overweight/obesity, it does not capture variations in fat and fat-free mass that are surely related differently to fitness. Nevertheless, it was recently shown that regardless the cut-off points, overweight/obesity assessed by BMI during childhood is a strong predictor of obesity and coronary heart disease risk factors in young adulthood, [48] which can lead to the importance of our data from a preventive point of view. Lastly, insulin was not measured, which we know that at these ages is very important. In fact, the clinical value of fasting glucose in the first decade of life has been questioned. Recent findings show that fasting glucose is weakly associated with clustering of cardiovascular disease risk factors in young people [49]. This can be explained by the fact the insulin resistance may increase before children display impaired glucose tolerance. In the very early stages of insulin resistance, the pancreas may compensate this metabolic disturbance by excreting higher quantities of insulin. Therefore, further studies should address and incorporate this analysis.

## Conclusions

In conclusion, this study adds data to the relation between muscular fitness and metabolic risk factors. Our data suggest that lower MF was associated with increased risk for obesity and metabolic risk in adolescent girls even after adjustment for age and maturation.

## Competing interests

The authors declare that they have no competing interests.

## Authors' contributions

JM gave contributions to conception and design of the study and carried out the data collection, analysis and interpretation of data. They also revised the draft; SV, CM, AG, CM and RS were involved in the analysis and interpretation of data and revised it critically for important intellectual content. SV, RS and JCR gave contributions to conception and design of the study he has been involved in drafting the manuscript and revised critically its content. All authors read and approved the final manuscript.

**Table 3:** Logistic Regression Analysis showing estimating results with Muscular Fitness as independent variable and BMI (normal vs. Overweight/Obese), Physical Activity (Active vs. Low Active) and MRS (Yes or No) as dependent variables.

		B	OR	95%CI	p
BMI (model 1)	MFO - REF		1		
	MF1	-0.63	0.53	0.24-1.19	0.13
	MF2	-1.29	0.27	0.12-0.64	**0.003**

MRS (model 2)	MFO - ref		1		
	MF1	-1.48	0.23	0.92-0.57	**0.02**
	MF2	-1.33	0.26	0.11-0.63	**0.03**

PAI (model 3)	MFO - ref		1		
	MF1	-0.169	0.84	0.38-1.84	0.67
	MF2	0.37	1.45	0.67-3.14	0.34

BMI - Body Mass Index

MRS - Metabolic Risk Score

PAI - Physical Activity Index
